# The Mechanical Basis of Memory – the MeshCODE Theory

**DOI:** 10.3389/fnmol.2021.592951

**Published:** 2021-02-25

**Authors:** Benjamin T. Goult

**Affiliations:** School of Biosciences, University of Kent, Canterbury, United Kingdom

**Keywords:** Memory, talin, mechanobiology, MeshCODE, brain, integrin, engram, cytoskeleton

## Abstract

One of the major unsolved mysteries of biological science concerns the question of where and in what form information is stored in the brain. I propose that memory is stored in the brain in a mechanically encoded binary format written into the conformations of proteins found in the cell-extracellular matrix (ECM) adhesions that organise each and every synapse. The MeshCODE framework outlined here represents a unifying theory of data storage in animals, providing read-write storage of both dynamic and persistent information in a binary format. Mechanosensitive proteins that contain force-dependent switches can store information persistently, which can be written or updated using small changes in mechanical force. These mechanosensitive proteins, such as talin, scaffold each synapse, creating a meshwork of switches that together form a code, the so-called MeshCODE. Large signalling complexes assemble on these scaffolds as a function of the switch patterns and these complexes would both stabilise the patterns and coordinate synaptic regulators to dynamically tune synaptic activity. Synaptic transmission and action potential spike trains would operate the cytoskeletal machinery to write and update the synaptic MeshCODEs, thereby propagating this coding throughout the organism. Based on established biophysical principles, such a mechanical basis for memory would provide a physical location for data storage in the brain, with the binary patterns, encoded in the information-storing mechanosensitive molecules in the synaptic scaffolds, and the complexes that form on them, representing the physical location of engrams. Furthermore, the conversion and storage of sensory and temporal inputs into a binary format would constitute an addressable read-write memory system, supporting the view of the mind as an organic supercomputer.

## Introduction

I would like to propose here a unifying theory of rewritable data storage in animals. This theory is based around the realisation that mechanosensitive proteins, which contain force-dependent binary switches, can store information persistently in a binary format, with the information stored in each molecule able to be written and/or updated via small changes in mechanical force. The protein talin contains 13 of these switches (Yao et al., 2016; Goult et al., 2018; Wang et al., 2019), and, as I argue here, it is my assertion that talin is the memory molecule of animals. These mechanosensitive proteins scaffold each and every synapse (Kilinc, 2018; Lilja and Ivaska, 2018; Dourlen et al., 2019) and have been considered mainly structural. However, these synaptic scaffolds also represent a meshwork of binary switches that I propose form a code, the so-called MeshCODE. The appreciation of such a network of switches and the machinery that controls them leads to a new hypothesis for the way the brain might be functioning.

**FIGURE 1:**
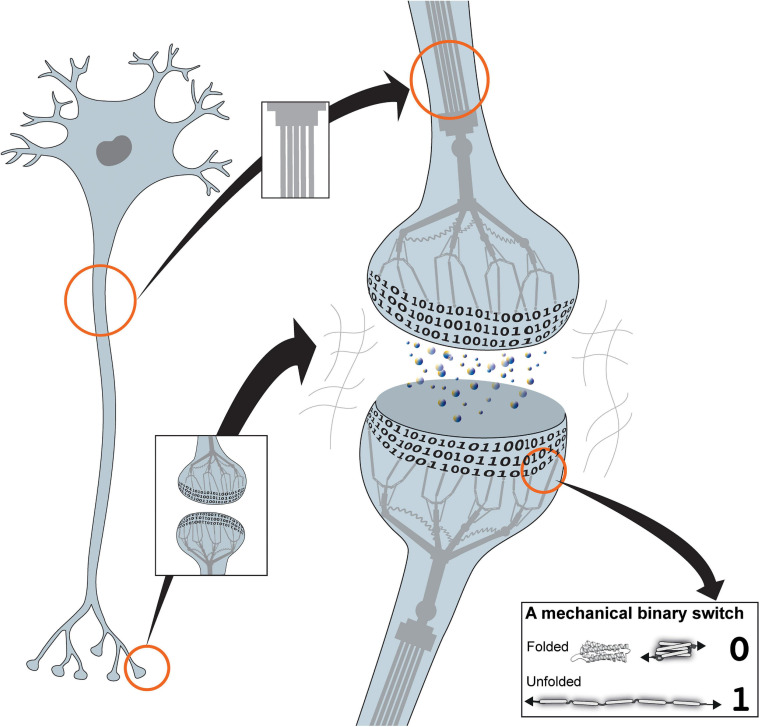
The mechanical cell. Cartoon of a neuron showing the binary coding that results from the hundreds of mechanical switches built into each synapse. The cytoskeleton is represented as a mechanical machine that operates these switches in response to neuronal activity, thus altering and updating the coding. Inset: example of a mechanical binary switch. These protein domains can be reversibly switched between two different states, folded “0” and unfolded “1.”

The MeshCODE array of mechanical switches^1^ would be operated by the cytoskeletal machinery, with synaptic signalling triggering the cytoskeleton to push and pull on these switches to constantly alter and update the coding in each neuron (Figure 1). Together, this mechanical layer would integrate with the chemical and ionic signalling layers to provide the basis for a new mechanism underlying information-processing and storage. In this scenario, electrochemical signalling between neurons would coordinate a network of trillions of mechanically operated switches, each able to store one bit of data, with action potential spike trains serving as the means to enter new information into this calculation. This mechanical coding would run continuously in every neuron, extending into every cell in the organism, ultimately amounting to a machine code coordinating the entire organism.

The concept of the mechanical computer described herein represents a new hypothesis for how the brain might be performing computation and postulates an addressable read-write memory mechanism. Such a memory mechanism would facilitate both the storage of data in an indexed, hierarchical structure and provide a basis for how information-processing machinery could call on this data as required. This novel concept for biochemical data storage and organic computation has similarities with computers, both old and new. These similarities span from the earliest mechanical computing machines (Babbage et al., 1973) through to solid state disks (SSDs) (Cornwell, 2012; Monzio Compagnoni et al., 2017) and the complex subroutines that enable complex computation.

## The Cell as an Organic Calculating Machine

The original concept of a digital programmable computer is attributed to Charles Babbage, whose “Analytical Engine” mechanical computer was first described in 1837 (Babbage et al., 1973). Early computers were built using mechanical components, complex machines of levers and gears that were used to perform calculations by turning gears and incrementing counters and ultimately output displays. The inputs initiate the calculation and the levers and gears crunch the numbers, and push and pull until the calculation is complete and the output returned. The programs that the Analytical Engine uses take the form of punched cards, in which the pattern of holes on the card run different parts of the machine. By changing the pattern of holes on the card, a different program will run to give a different calculation and obtain a different output.

There are considerable similarities between a mechanical computer and a cell. Each cell contains a series of levers, pulleys and gears in the form of its cytoskeleton. The cytoskeleton is an incredibly complex, dynamic network formed of three major classes of filaments; actin, microtubules, and intermediate filaments (Fletcher and Mullins, 2010). These filaments can assemble and disassemble rapidly and robustly in response to cellular signals, and the interplay between them is complex. The cytoskeleton can be used to generate forces, with motor proteins pushing and pulling on actin and microtubule filaments, exerting forces on to specific targets. These filaments can also serve as railroads to transport cargos to precise locations in the cell. There are hundreds of cytoskeletal regulators that control these networks with the precise linkages, filaments, and adapters determined by the programme that cell is running.

Almost all cells in our bodies rely on cell-adhesion molecules, that adhere the cell to adjacent cells and/or the surrounding meshwork of proteins called the extracellular matrix (ECM). The adhesions to the ECM, mediated by the integrin family of ECM receptors, serve as information-processing centres, able to feel the surrounding environment and instruct the cell how to function (Iskratsch et al., 2014). The cytoskeleton is wired up to the cells adhesions, to the nucleus and to all the organelles in a highly ordered (but dynamic) manner. Once a cell is established in its environment, the cytoskeleton is maintained under tension but is not constantly generating large forces or battling against itself. This homeostasis is achieved when all the forces and tensional restraints are balanced in the system, at which point it is said to be under tensional integrity or “tensegrity” (Fuller, 1961; Ingber, 1997; Reilly and Ingber, 2018). In a muscle, the actin filaments and myosin motors are highly organised to enable the generation of forces and motion. In a similar fashion, the cytoskeleton in the brain is ordered (Hotulainen and Hoogenraad, 2010; Leterrier et al., 2017) which I suggest connects all the synaptic adhesions together via dynamic mechanical linkages.

### Talin

**FIGURE 2:**
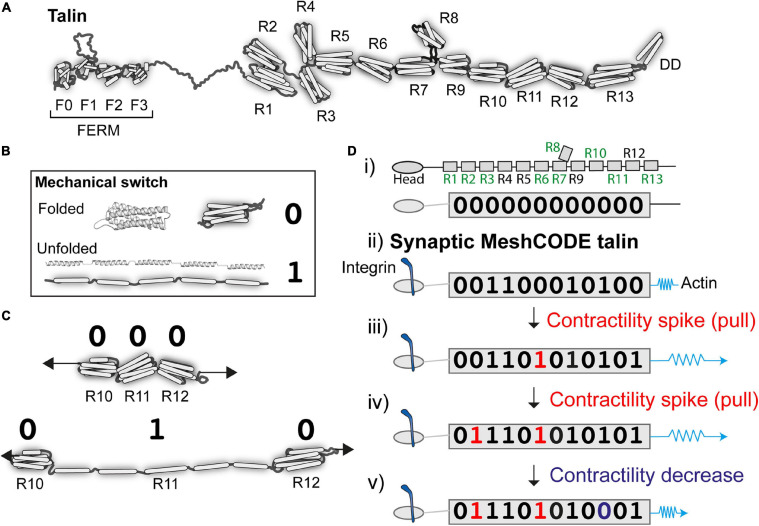
Talin as a memory molecule. **(A)** Talin is comprised of an N-terminal FERM domain that binds to integrin, connected to 13 helical bundles, the talin rod domains R1-R13. **(B)** A mechanical binary switch. One talin helical bundle is shown. Under tension each bundle can exist in two thermodynamically stable states, folded “0” and unfolded “1” and can be switched back and forth between these states via mechanical force. **(C)** Cartoon of three rod domains going from 000 to 010 in response to one contractility spike by the cytoskeletal force-generation machinery. **(D)** A talin molecule as a binary string, the nine vinculin binding sites are labelled in green (i) in the absence of force the 13 rod domains are all in the folded “0” state; (ii) upon adhesion formation each talin is tethered between the integrin:ECM and F-actin, and adopts a specific switch pattern; two contractility spikes (shown as a blue spring) result in two of the switches switching (iii and iv), whereas a decrease in contractility resets one switch back to 0 (v). The exact order of switching and the resulting switch state will depend on the system.

**FIGURE 3:**
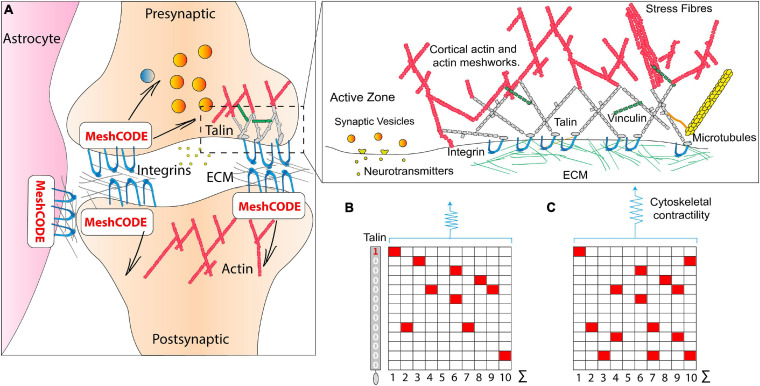
The MeshCODE. **(A)** Schematic diagram of a tripartite synaptic junction. The integrin adhesion complexes and MeshCODEs in the presynaptic and postsynaptic zones, and in a neighbouring astrocyte are shown. The synapse is encapsulated by a specialised ECM that protects the mechanical environment of the MeshCODEs. Integrin and talin in one MeshCODE are shown connected to the actin cytoskeleton. (Right) Enlarged view of the highlighted region showing the MeshCODE intricately wired up to the cytoskeleton. Only the integrin-talin-actin adhesion core complex is shown, but up to 250 different proteins decorate this core dependent on the switch patterns of the talin scaffolds. The protein vinculin (green) binds to nine of the unfolded talin switch domains, stabilising them in the unfolded state and reinforcing the cytoskeletal connections. **(B)** A schematic of an array of ten talin molecules with the 13 switches arranged vertically. White, 0; red, 1. **(C)** Perturbations to the system result in changes in cytoskeletal contractility that alter the pattern of 1s and 0s written in the MeshCODE and its resultant output in a defined way.

The major linkage between the integrin-ECM connections and the cytoskeleton is the protein talin (Calderwood et al., 2013; Klapholz and Brown, 2017; Figures 2, 3). Talin is perfectly positioned to respond to changes in forces, both from outside and inside the cell, and has emerged as a master mechanosensor in that it can sense these forces and convert them into biological signals (Klapholz and Brown, 2017; Goult et al., 2018). Each adhesive structure contains hundreds of talin molecules (Changede et al., 2015) all connected to the integrins, each other, and the cytoskeleton through direct and indirect connections to actin (Hemmings et al., 1996; McCann and Craig, 1997; Gingras et al., 2008), creating a complex array of talin molecules (Figure 3A). Onto this integrin-talin-actin core complex, numerous additional proteins assemble (Winograd-Katz et al., 2014; Horton et al., 2015) forming distinct, tissue specific signalling complexes (Harburger and Calderwood, 2009).

There are two talin isoforms, talin-1 and talin-2 (Senetar and McCann, 2005; Debrand et al., 2009; Gough and Goult, 2018) expressed in the brain; both have the same domain structures (Gough and Goult, 2018) but subtly different mechanical properties (Austen et al., 2015). Each talin molecule contains 13 force-dependent binary switches (Goult et al., 2013; Yao et al., 2016; Figure 2A). These talin domains, can be reversibly switched between two thermodynamically stable states, “folded” and “unfolded,” using mechanical force (Figure 2B). One of the key discoveries that leads to the MeshCODE theory is that the switches in talin exhibit mechanical hysteresis (Yao et al., 2016) meaning that, provided the molecule is maintained in a mechanical linkage between the integrin-ECM and the actin cytoskeleton, both the folded and unfolded states of each domain are thermodynamically stable (Figure 2B). This imparts a novel function on talin in molecular memory as each talin molecule can be imprinted with a persistent pattern of switch states as a specific outcome of the forces that have acted upon it (Figure 2C). The conformational state of each switch determines which signalling molecules are recruited, thereby providing different instructions to the cell as a function of force, enabling talin to serve as a Mechanosensitive Signalling Hub (described in Goult et al., 2018). As the environment changes, such as when a cell migrates, these switches detect these changes enabling the cell to respond appropriately. Most current models envisage talin as a rope in a “tug-of-war” between extracellular forces, and those generated by the cells force-generating machinery serving as a “molecular clutch” that enables mechanotransduction and cell migration (Mitchison and Kirschner, 1988; Elosegui-Artola et al., 2016; Swaminathan and Waterman, 2016). Proteins such as vinculin, which bind to nine of the unfolded talin switches, can further stabilise the unfolded state (Carisey et al., 2013; Yao et al., 2014), limiting refolding and reinforcing these connections.

#### Talin, the Data Molecule of Life

Every animal known to humankind has the same 13 switches in talin (Senetar and McCann, 2005; Goult et al., 2013), and the high conservation of switch pattern suggests a role that is explicitly dependent on the order of the string of switches. The best way to visualise such a role is to consider the scenario where the extracellular environment is built in such a way that it presents a mechanically stable, predictable environment. In this scenario, the talin switches are no longer required to sense the extracellular environment as that is, to all intents and purposes, constant. Instead, the cell can use its force-generating machinery to operate these mechanical switches and in doing so write data into the adhesions (Figure 2D).

This has profound implications for data storage in animals, as it means that the cell can use the talin switches to store information. Adhesions in controlled, predictable environments can adopt the role of data-storage devices that store information in a manner controlled by the system. I would like to propose that this complex meshwork of switches operates as a code, the MeshCODE outlined here. To simplify the nomenclature of the switches in this view of talin it is easier to regard the “folded” and “unfolded” states as “0” and “1,” respectively, to better reflect that it is data that is being stored in the talin molecule (Figure 2).

### Exquisite Calculation in a Single Cell

As a result, the cell is a mechanical computer with all of the architecture necessary for computation. The cytoskeleton serves as the levers and gears that perform the calculation, and the MeshCODE adhesions provide a multifunctional system that can be used to perform calculations and serve as a memory storing the results in the conformations of the switches. An input signal, be it an extracellular signal activating a receptor, a change in physicality, or excitation by a chemical or electrical signal, perturbs the balanced state of the cell, switching the “computer” on, thereby triggering changes in the cytoskeleton as it seeks to return to homeostasis. Key to maintaining the mechanical balance of the cell are the 20 members of the Rho-family of GTPases (Lawson and Ridley, 2017). These crucial components of the signalling networks coordinate the contractility and behaviour of the actin cytoskeleton (Jaffe and Hall, 2005) and their role in controlling synaptic development and plasticity is established (Threadgill et al., 1997; Tolias et al., 2011; Ba et al., 2013). There are 80 activators [guanine nucleotide exchange factors (GEFs)] and 70 inactivators [GTPase-activating proteins (GAPs)] (Bos et al., 2007; Bagci et al., 2020) of the Rho family of proteins, many of which are located at synapses (Wilkinson et al., 2017). The balance of these opposing regulators enables the cellular mechanics to be spatially and temporally modulated by a multitude of signalling pathways. Changes in architecture and contractility push and pull on the adhesions which the MeshCODE theory predicts will result in reproducible alterations in the binary switches. When the calculation is complete, homeostasis is restored. At this end point, the conformations of the switches in the adhesions are altered and the array of 0s and 1s reflect the outcome of the calculation. The large adhesion complexes that assemble on the talin scaffold as a function of the altered switch patterns (Goult et al., 2018) stabilise the patterns and dictate the signalling response.

The appearance of talin at the dawn of multicellularity allowed cells to store information persistently by writing to each talin molecule like a computer writes to a disk. As well as serving as a memory, these switches also coordinate cell signalling (Goult et al., 2018) and provide a way to control the reading of the genome from the periphery of the cell (Elosegui-Artola et al., 2016).

### Organic Calculation in the Brain

The brain is a colossal cell signalling machine with a trillion cells all communicating with each other, leveraging the organic calculating power of each cell. Synapses are the perfect system for optimised cell signalling between connected cells, and there are approximately 100 trillion synapses in the brain (Pakkenberg et al., 2003) transmitting signals between neurons, to give rise to brain activity. The specialised architecture of synaptic junctions juxtaposes the presynaptic and postsynaptic neurons to form the synaptic cleft (Mayford et al., 2012; Asok et al., 2019), an arrangement supported by astrocytes which coordinate synaptic maintenance and plasticity, and microglia (Perea et al., 2009; Wu et al., 2015; Figure 3A). Each synapse is scaffolded by adhesions located around the edge of the synapse (Dityatev et al., 2014; Park and Goda, 2016; Kilinc, 2018; Lilja and Ivaska, 2018); these adhesions involve the binding of integrins to ECM components, such as laminin and fibronectin (Levy et al., 2014; Figure 3A). Integrin adhesion complexes are required for synaptic plasticity and memory (Chan et al., 2003), and consolidation of long-term potentiation (Kramár et al., 2006) and the ECM has been linked to learning and memory previously (Dityatev et al., 2010; Levy et al., 2014). However, a role for adhesion complexes in the storage of data has not been considered previously.

The brain is soft, which provides the perfect, protective environment for each neuron to tightly control its own mechanical environment independently, building its own ECM niche around each synapse (Levy et al., 2014), with the surroundings serving to isolate the neuron from external mechanical forces. The MeshCODE theory proposes that tight regulation of the mechanical environment of each synapse would enable mechanical computation to occur with meticulous precision. The ECM surrounding each synapse is subject to extensive maintenance and remodelling during synaptic function (Wlodarczyk et al., 2011; Kurshan et al., 2014; Levy et al., 2014; Ferrer-Ferrer and Dityatev, 2018), and it seems reasonable to postulate that this maintenance of the ECM might ensure that the synapses mechanical environment is tightly regulated and predictable.

Therefore, in the MeshCODE framework, these synaptic scaffolds provide the capacity to write data into the synapses themselves in the extensive arrays of binary switches that are located in both the pre-, and postsynaptic side of each synapse as well as in the supporting astrocyte cells (Figure 3A). The capacity to store information in every synapse, with the potential to orchestrate the flow of information through that synapse, makes the synapse a complex computational device.

### How Would a Mechanical Code Be Read?

The key to any code is a read-out mechanism and the mechanical code outlined here leads to a number of hypotheses for how such a coding in the brain would be read. This section discusses how MeshCODE driven adhesion signalling might work in the context of a neuron, with the purpose being to demonstrate the feasibility and broad applicability of the theory to well-established neuronal processes.

**FIGURE 4:**
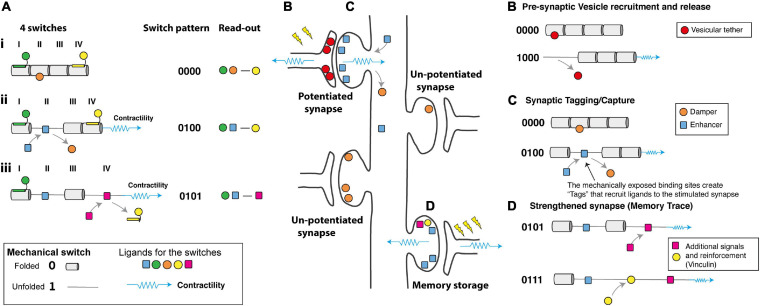
How would a mechanical code be read? **(A)** Schematic diagram representing four mechanical switches labelled I–IV, (Left) each switch has the potential to bind different ligands in its folded, 0 and unfolded, 1 states. (Middle) The switch pattern is shown as a binary string. (Right) The ligands decorating the switches present a “read-out” mechanism as the complexes formed will depend on the mechanical coding. **(i)** at low force the green, orange, and yellow ligands engage the MeshCODE on switches I, II, and IV. **(ii)** Contractility (shown as a blue spring) switches one domain (here domain II) from the 0 to 1 state which drives a switch in binding partners, displacing the orange ligand and recruiting the blue ligand. The overall signalling complex and read-out is altered. **(iii)** Further contractility switches a second domain (here domain IV) further altering the coding and the proteins that are recruited to the synaptic scaffolds. Proteins, like vinculin, which bind the unfolded state of a switch lock it in that conformation and limit its ability to refold. **(B–D)** A cartoon of a neuron with four synapses in different states showing hypothetical ways a mechanical code might be read. Contractility as a result of synaptic signalling causes alterations to the MeshCODE switch patterns on both sides of a stimulated synapse. **(B)** in the pre-synaptic terminal these switch patterns might regulate the probability of release of docked vesicles by specific switches controlling this process via a hypothetical interaction with a key regulator of synaptic firing. **(C)** Synaptic stimulation triggers contractility in the post-synaptic region that specifically alters the switch patterns in that stimulated synapse. The altered switch patterns create “Tags” that recruit proteins (one is shown as a blue square) specifically to that synapse that enhance long-term potentiation. Switching might also displace proteins that dampen potentiation (orange circle) which then diffuse away. **(D)** Future signals through that synapse can trigger additional switch pattern changes and alter the binary coding in that synapse, the protein signals recruited, etc., in a dynamic re/writable way, changing the threshold of each synapse in that neuron. These changes to the synaptic adhesions as they grow and shrink dynamically in response to stimuli form the memory trace.

The contractility-dependent control of integrin-adhesion complexes is a well-studied phenomenon in the cell adhesion field (Vicente-Manzanares and Horwitz, 2011; Sun et al., 2016; Gauthier and Roca-Cusachs, 2018; Han et al., 2019). Adhesion complexes grow as forces are exerted on them, altering their composition and signalling outputs and mechanical forces acting on talin are essential to drive this process (Rahikainen et al., 2019). Each synaptic adhesion would serve as a discrete signalling hub, altering its signalling as a function of the mechanical signals received. Following each synaptic signalling event, the neuronal cytoskeleton would contract briefly and precisely generating these mechanical signals and altering the switch patterns at that site. As a result, the code would be read by the ligands that engage the different switch states (Figure 4A) with the read-out being the relative (re)distribution of all the ligands engaging the entire array of switches defining the output.

The location of switches in the pre- and post-synaptic scaffolds, coupled with the ligands available to bind in these different structures, suggests potential mechanical explanations for a number of recognised processes of synaptic regulation including; (i) dynamical regulation of the probability of vesicular release (Dürst et al., 2020; Figure 4B), (ii) synaptic tagging and capture (Frey and Morris, 1997; Martin et al., 1997; Martin and Kosik, 2002; Kelleher et al., 2004; Sajikumar and Frey, 2004; Redondo and Morris, 2011; Nonaka et al., 2014; Figure 4C), (iii) inverse synaptic tagging (Nonaka et al., 2014), (iv) synaptic competition (Fonseca et al., 2006; Cancedda and Poo, 2008; Ramiro-Cortés et al., 2014; Sajikumar et al., 2014; Figure 4D), (v) spike-timing dependent plasticity (Campanac and Debanne, 2008; Markram et al., 2012; Pang et al., 2019) and (vi) the capture of temporal information.

#### Hypothesis: Synapses Are Activated and Deactivated Transiently in Response to Signals

The presence of switches in the synapses raises the possibility that certain MeshCODE patterns might transiently activate, or deactivate, transmission through specific synapses. On the pre-synaptic side, the strength of individual synapses within a single axon are regulated by changes in the probability of vesicular release (Dürst et al., 2020). Tightly regulated changes in the talin switch conformation at individual synapses might provide a molecular mechanism for this changing probability (Figure 4B). The actin cytoskeleton has been shown to be important for neurotransmitter release (Doussau and Augustine, 2000; Kneussel and Wagner, 2013), as has the requirement of force generation at these sites (Jung et al., 2016), and a role for talin in presynaptic function has also been reported (Morgan et al., 2004). In the pre-synaptic terminal alterations in certain switch patterns, dynamically exposing and disrupting binding sites in close proximity to the active zone, might regulate the number of docked vesicles, or control the probability of release of docked vesicles via hypothetical interactions with key regulator(s) of synaptic firing (Figure 4B). The probability of each synapse firing would be updated by the mechanical signalling providing a mechanism for synapses to be switched on and off transiently on demand, potentially contributing to the homeostatic synaptic scaling (Turrigiano, 2008) of the neurons output.

#### Hypothesis: A Mechanical Basis for Synaptic Tagging?

A well-known, but not fully understood, phenomena is the process of “**synaptic tagging and capture**,” where following activation of a synapse, that synapse is marked in such a way that proteins are recruited directly to that synapse (Frey and Morris, 1997; Martin et al., 1997; Martin and Kosik, 2002; Kelleher et al., 2004; Sajikumar and Frey, 2004; Redondo and Morris, 2011; Nonaka et al., 2014; Figure 4C). As a synapse is stimulated, then the MeshCODE theory predicts that the stimulated synapse undergoes localised contractility thereby altering the switch patterns at that site. Certain switch states would be generated that expose novel binding sites in that stimulated synapse only. These “Tags” (Figure 4C) would serve to recruit proteins to that synapse and it is possible that these switches provide the basis for a dynamic form of synaptic tagging. It is already established that alterations in talin conformation cause global relocation of cellular factors (Lee et al., 2013; Elosegui-Artola et al., 2017; Haining et al., 2018). Furthermore, it is also possible that proteins that inactivate synapses, and/or transcriptional regulators, might be bound to the 0 state of the switch which upon the switch unfolding would be released from the newly stimulated synapse to diffuse away (Figure 4C). This displacement of signals from a stimulated synapse would provide a mechanism for “**Inverse synaptic tagging**” (Nonaka et al., 2014); “inverse tag” proteins would be displaced from stimulated synapses and accumulate at unstimulated synapses where more of that switch is still in the 0 state.

As protein levels in cells are under tight control via transcriptionally regulated programs, these switches would result in the global relocalisation of proteins in the neuron. If proteins are recruited to one synapse following stimulation (Figure 4C), then those same proteins would be depleted from other synapses leading to a basis for “**synaptic competition**” (Fonseca et al., 2006; Cancedda and Poo, 2008; Ramiro-Cortés et al., 2014; Sajikumar et al., 2014) where the strengthening of one synapse affects the stability of other synapses in that cell. The number of synapses, and the number of scaffold proteins in each synapse will be tightly regulated, and therefore an exact number of mechanical switches will be distributed across each neuron, and throughout the brain. As each switch state can recruit one signalling molecule then as the number of switches in one state increases in a stimulated synapse more of that signalling molecule will be recruited to that site thereby dynamically altering the concentration of molecules at that adhesion/synapse and depleting that molecule from the rest of the neuron and the other unstimulated synapses. This mechanical coordination between synapses, with stimulation of one synapse recruiting and displacing plasticity-related proteins from that site, thereby directly effecting the proteins available to bind at other synapses provides a mechanism for potentiated and depressed synapses to interact in a synergistic manner. This synchronisation would enable the entire neuron to behave as a mechanically coordinated system of synapses where input signals to a synapse can dynamically alter the activity of each synapse in the neuron (and by extension the neurons it is communicating with), providing a way to establish long-term plasticity and orchestrate the flow of information transmitted by the cell.

Overtime this synaptic tagging leads to remodelling of the synapse, driven by the recruitment of newly transcribed proteins to the stimulated site (Frey and Morris, 1997; Tonegawa et al., 2015). Remodelling of the actin cytoskeleton occurs at stimulated synapses (Hotulainen and Hoogenraad, 2010; Basu and Lamprecht, 2018), which not only enhances that synaptic connection but also strengthens the mechanical linkages mediated through the actin cytoskeleton emanating from that site. Nine of the unfolded switch domains in talin recruit the protein vinculin, which is well known as a means that cells reinforce integrin-talin-actin connections anchoring actin to the adhesion site (Humphries et al., 2007; Carisey et al., 2013) and also for locking the mechanical switches in the unfolded state (Yao et al., 2014).

#### Hypothesis: Contractility-Dependent Control of Integrin Adhesion Complex Dynamics Is the Way That Neurons Store Information

It might be that the Synaptic tagging observed in synapses (Frey and Morris, 1997; Martin et al., 1997; Martin and Kosik, 2002; Kelleher et al., 2004; Sajikumar and Frey, 2004; Redondo and Morris, 2011; Nonaka et al., 2014) is actually a similar process to the contractility-dependent growth of integrin adhesion complexes seen in other cells (Vicente-Manzanares and Horwitz, 2011; Sun et al., 2016; Gauthier and Roca-Cusachs, 2018; Han et al., 2019). The adhesions would grow in stimulated synapses as forces are exerted on them and atrophy in unstimulated synapses, with the cumulative effect that overtime these changes in switch pattern at stimulated synapses would lead to morphological changes in the shape and size of the synapses as they remodel altering the signalling output of each site. This process would be explicitly dependent on the talin switch patterns that dictate which molecules assemble to form each adhesion complex. The engagement of the cytoskeleton with the talin molecules directly, and indirectly through proteins such as vinculin, will alter as the switch patterns change, and these engagements will (i) help maintain the switch pattern and (ii) alter where and how future contractility spikes exert tension on talin (Atherton et al., 2015; Kumar et al., 2016; Ringer et al., 2017) and the meshwork in general, and (iii) determine the signalling molecules recruited to that site. It is possible that the patterns of the switch states and the specificity of ligands binding to them, are further regulated by post-translational modification; specific patterns could be “write protected” by locking the domains in those conformations by phosphorylation or other modification.

These synaptic adhesions would be serving as large information-processing centres, with the many proteins recruited to, and interacting on, the patterns of switches stabilising the patterns and encoding/decoding the information. The complexes that assemble on the patterns (Figure 4D) and the signalling outputs that result will vary depending on the expression levels of proteins, and their distribution, in that neuron, but in each case, they will be assembled on the integrin-talin-actin core complex dictated by the mechanical signalling across the whole cell. Furthermore, adhesion complexes also transmit mechanical signals to the nucleus (Graham and Burridge, 2016), and can induce modular gene expression patterns in response to both physical and geometric constraints (Engler et al., 2006; Jain et al., 2013; Jahed et al., 2014; Elosegui-Artola et al., 2016; Cooper and Giancotti, 2019), a process referred to as mechanotransduction. Epigenetic tagging and alterations in gene expression are required for memory system consolidation (Lesburgueres et al., 2011; Kim and Kaang, 2017) and it might be that mechanotransduction through these synaptic adhesion complexes contributes to such mechanisms.

Subsequent stimulation of that synapse would result in more contractility changes that further alter the switch patterns in the MeshCODE dependent on the current switch patterns at that site and the cytoskeletal connections that formed. Further contractility might unfold more copies of the same switch domain that was described above (Figure 4C), exposing more “tags” and recruiting more of the ligand that binds to that site further depleting it from other synapses. However, if many copies of that switch are already in the unfolded state, or if the cytoskeletal connections have altered, then additional contractility would trigger different switches to unfold, recruiting and displacing other molecules, causing further remodelling of the adhesion complexes and redistribution of proteins throughout the neuron (Figure 4D).

#### Spike-Timing Dependent Plasticity and a Mechanism for Capturing Temporal Information

This complex mechanical signalling would provide a means for capturing temporal information and a basis for **spike-timing dependent plasticity** (Campanac and Debanne, 2008; Markram et al., 2012; Pang et al., 2019); multiple spike trains of signals stimulating that synapse would result in multiple contractility events with the result that additional patterns of 1s and 0s would form dictated by the frequency of the stimulations (Figures 3B,C). Different spike-timings would trigger different patterns giving rise to different neuronal responses, and overtime these patterns would get consolidated as the neuron remodels.

Mechanical switches provide a way to rapidly capture information on a fast timescale, requiring only changes to a proteins shape. These shape changes alter the binding sites available at the synapse and enable other proteins to coalesce onto the talin scaffold to alter the dynamics and composition of each synaptic adhesion. The exposed switch states only need to persist long enough for ligands to engage the newly exposed binding sites (or be displaced from the disrupted binding sites) to form the adhesion signalling complexes that assemble onto the talin scaffold as a function of its switch state (Goult et al., 2018). Our research has shown that the switch patterns are stable under force for many minutes (Yao et al., 2016), which provides a temporal element to the switch signal persistence, if it recruits a ligand (or is modified) it will persist, otherwise that switch state will be more dynamic and the information more transient. In regions of the brain where there are many inputs, this dynamic nature might be advantageous. Without systems in place to stabilise these conformations, this type of information would be quick to write, but short-lived. This speed of writing might allow the brain to rapidly encode sensory and temporal information originating from outside of the organism, integrate it into the coding, and transmit signals to other regions of the brain for processing.

#### Hypothesis: This Binary Information Controls the Thresholding of Synaptic Output

However, with the systems in place to stabilise these conformations, integrin adhesion complexes are stable and can persist for a long time with the information stored in them explicitly dependent on the switch patterns of the talin molecules at the core. Each stimulation would alter the switch pattern and over time these patterns would be consolidated as the adhesions at each synapse, the synapse and the whole neuron remodel tuning the strength of each of the synaptic connections. The signalling at each synapse would be tuned by the adhesion signalling complexes around its periphery. Competition between synapses due to the changing distribution of mechanical tags within the same post-synaptic neuron tightly controlled by inputs from pre-synaptic neurons provides a mechanical basis for the synchronisation of the entire neuron, enabling the whole neuron to function as a computational system, providing a mechanical basis for the experimentally observed models of long-term engram formation elegantly shown by others (Govindarajan et al., 2006). These switches would drive structural remodelling of the entire cell to cause system level changes to the architecture and properties of that neuron and the connections it makes, changing the thresholds of each synapse in the neuron and thus the flow of information. As each neuron can have more than 10,000 synapses, it might be that these can be turned on and off with high specificity enabling precise stimulation of different synapses and neuronal circuits directing the signals to specific synapses for data storage. MeshCODE operations that allosterically enhance or dampen synaptic signalling (Figures 4A–C) would ensure that data is directed to the right memory modules. Activation of one synapse in a neuron would lead to alterations in other synaptic MeshCODEs in that neuron, mediated mechanically via the cytoskeleton and via diffusion of soluble factors as a result of neuronal signalling and this would be dependent on all the other nerves communicating with it. These changes would dynamically alter both the stored information and the transmission through the entire neuronal network.

**FIGURE 5:**
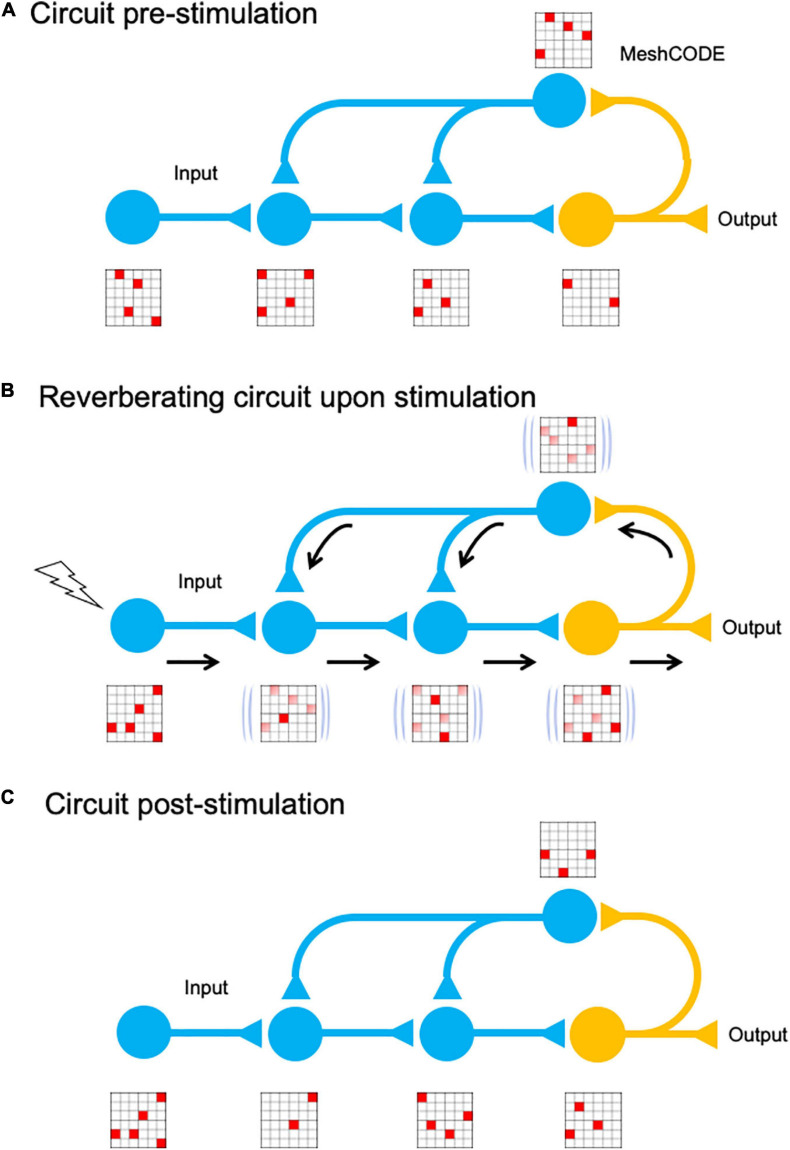
MeshCODE updating. Cartoon of a simplified neural circuit comprised of an input neuron, which inputs into a three-neuron circuit (blue) that is wired up to an output neuron (orange). A schematic of a MeshCODE is shown for each neuron (for clarity just a 6 × 6 array is shown). White, 0, Red, 1. **(A)** At rest, the MeshCODEs throughout the circuit are in a specific pattern of 1s and 0s. **(B)** Stimuli input into the system perturbing the equilibrium state across the entire circuit. The calculation occurs as the cytoskeletal alterations cause changes to the coding of the system. The relevant output from the circuit is transmitted. **(C)** Following the input, and the resulting calculation, the MeshCODEs in that circuit are altered in a way that encodes information.

The ability for re-writable, long-term storage of information in the conformational patterning of the MeshCODEs, means data storage in the brain would be encoded in a binary format written into each and every synapse (Figure 5). Each input signal alters the MeshCODE patterns in that synapse and other synapses in the neuron. As this neuron would be communicating with other neurons, part of this calculation would involve signals being emitted through specific synapses, switching on such calculation in adjacent cells (Figure 5). In each of these synaptic transmissions, the binary coding of the MeshCODEs across that synapse would be altered. As a result, the re-balancing and return to a metastable end state would need to occur across entire circuits. The pattern of 1s and 0s in each synapse across the entire network of neurons would be inextricably linked, all running the same mechanical code.

### Machine Code

Every MeshCODE of every synapse of every neuron of every circuit would contain information representing the current state of the organism. The coding would therefore represent a type of machine code the organism is using. Like most computer-machine codes, in the MeshCODE framework, the brain would be using a binary format. Every synapse in the brain would be written with the code, which would be constantly changing in response to the signals from the system. Some circuits would be stable, and others would be rapidly changing. Furthermore, MeshCODEs are found at all places where cells engage the ECM, so having a machine code running in every cell in the organism would hint at a unifying theory of cell communication, which provides an instruction set for every cell to work in synchrony. Like computer machine code, without the necessary parsers to correctly decode the symbols, the code is unreadable to an outsider and would look like just pulses of electrical activity, triggering alterations to vast strings of 1s and 0s. However, once the language of the code is understood this information might be decipherable and reveal an unimaginable level of communication.

#### Mathematical Representation – Overcoming the Binding Problem

Stimuli acting on sensory receptors result in action potential spike trains that transmit external information to the brain (Perkel et al., 1967; Strong et al., 1998; Fellous et al., 2004). Since every information sender and receiver in the organism is running the same operating system, action potential spike trains would serve as the vehicle to enable the transfer of information encoded in a way that allows the updating of the coding of the sender and receiver (Shannon, 1949; Gallistel and King, 2009). A machine code provides a mechanism for the brain to integrate all sensory inputs and outputs into a single coherent whole, which can be processed and compiled into a mathematical representation of the entire life of the organism. For example, sensory input from vision does not just function in isolation, but inputs into the calculation that contains every other sensory input contextualised in the entire learned experience. All of these different cues are processed and form part of a unified cohesive experience, and a mathematical representation would provide an explanation for how all this information is bound together.

MeshCODEs provide a framework for organic calculation in the brain and the potential for every synapse to contain the coding for the current best information available to the organism for immediate usage. As a result, the reason the brain can process and react so quickly despite electrochemical transmission being slow (eight orders of magnitude slower than in a computer) would be because each neuron and motor synapse is primed with information that is being constantly updated. The calculation is performed predominantly in the brain but distributed across the whole organism.

## Implications of a Physical Location for Data Storage

In the previous sections I have presented the MeshCODE theory, and how mechanical switches provide a mechanism for how the brain could convert diverse inputs into a physical binary format in a way that controls the flow of information by tuning the thresholds of each synaptic connection. In this section, I discuss the implications that a physical location for data storage has for how the brain might be performing computation and propose that the MeshCODE framework provides a basis for an addressable read-write memory mechanism. I then consider the practicalities of storing terabytes of physical data (Box 1), and how data might be allocated and stored in the brain in a way that allows rapid recall.

**Box 1. What is the memory-storage capacity of the human brain in the MeshCODE framework?**

This box provides a “back of the envelope calculation” for how much memory-storage might be possible in the MeshCODE framework* based solely on how many mechanical binary switches might be present in the brain and the assumption that each binary switch could store 1 bit of information.

Talin has 13 binary switches, but for simplicity of numbers let us assume that each talin contains eight switches, or eight bits of information = 1 byte per talin.

If we assume 100 talins per synapse (likely to be more), then each synapse might contain 800 switches** (100 copies of each of the eight talin switches) with the capacity to encode 100 bytes of information.

If we assume 100 trillion synapses in the brain, then the MeshCODE data storage would be 100 bytes per 100 trillion synapses = 10,000 trillion bytes = 10 petabytes of global brain memory capacity***.

*How much data could MeshCODE arrays in the cortex hold?*

If we consider the cortex as the location of long-term memory information, what is the storage capacity of this region? If each pyramidal cell has 10,000 synapses with 100 bytes/synapse, each pyramidal cell can store ∼1 MB. If 100 pyramidal cells form a minicolumn, then each minicolumn can contain 100 MB. There are 100 million minicolumns, so there is a potential storage capacity of 100 terabytes in the cortex.

*Only talin is considered here, but there are many other proteins in the meshwork that also contain mechanical binary switches that will contribute to the global information content in the brain.

**The read-out of the code might be the weighted average of all the individual states of each switch within each synapse as that is what alters the relative levels of activity across the entire neuron.

***The memory capacity has potential to be significantly larger as all cells in the body (as well as astrocytes and glia in the brain) also have MeshCODE capacity. It is possible that each cell is encoded with updated memory information as part of the brain’s computation.

### Hypothesis: The Brain Cortex Is an Array of Memory Modules

**FIGURE 6:**
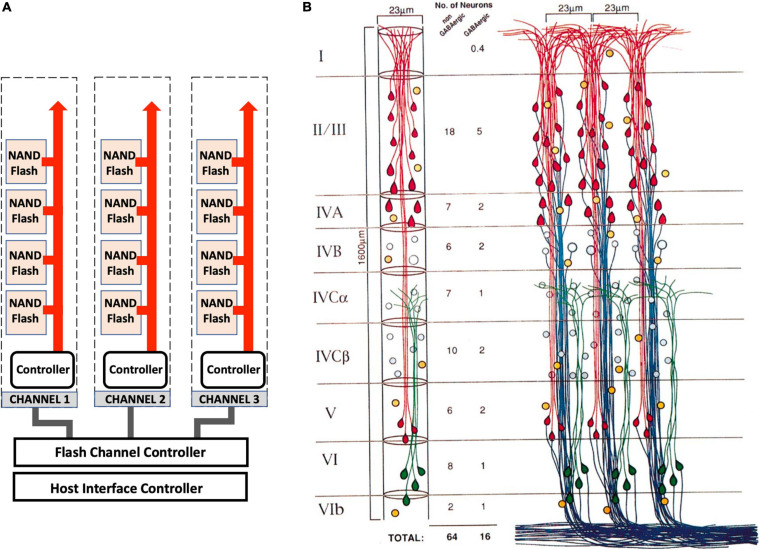
Architectural similarities of memory storage *in silico* and *in vivo*. **(A)** A high-capacity SSD storage device is incredibly complicated, but its general architecture is arranged into a logical repeating structure of pages, arranged into blocks that are arranged into memory modules (NAND Flash). These hierarchical structures are linked to channels that connect each memory module to a memory controller that controls the data coming in and out of the drive and directs current to the relevant pages and transistors for data read-write. **(B)** The cortex shares a similar logical architecture. Three cortical columns are shown that are equivalent to the SSD channels. These columns contain layers (I–VI) that are comprised of minicolumns (the memory modules equivalent to the NAND Flash), which are comprised of circuits of pyramidal neurons (the blocks) that contain tens of thousands of MeshCODEs in the synapses and dendritic spines (the pages). Each column is linked with read and write channels back to the hippocampus. Adapted with permission from Peters and Sethares (1996).

A major requirement of any computational device is the capability for long-term memory storage and retrieval (Gallistel and King, 2009; Gallistel and Balsam, 2014). Memory associations and long-term memory are thought to be stored in the cerebral cortex (Kandel et al., 2014). The architecture of the cortex is remarkably logical, made of distinct sub-regions that control different processes (Zingg et al., 2014). The arrangement of neurons in each sub-region of the cortex is highly ordered, arranged into 1–2 million cortical columns (Peters and Sethares, 1996; Mountcastle, 1997; Figure 6). Each cortical column has a logical structure, containing many pyramidal neurons each with more than 10,000 synapses (Spruston, 2008). Approximately 100 pyramidal neurons arrange into minicolumns, which wire up to form the cortical columns. In this arrangement there are many synapses and dendritic spines linked together in highly ordered arrays, which might form memory modules. These memory modules are connected to the hippocampus allowing information to travel back and forth between the cortex and the hippocampus. If each MeshCODE in each synapse in each column can be written to specifically, then this would represent a huge “disk” that would provide a mechanism for physical storage of data. This layered arrangement and its intricate connectivity patterns has striking similarities to the architectural layout of a SSD (Cornwell, 2012; Monzio Compagnoni et al., 2017; Figure 6).

Information could thus be written into such an array in a similar fashion to how data is stored on an SSD in computers. Synaptic connections that have the highest transmission might define the major connections through which the signal is passed most often. These strong wiring links might wire up the neurons into precisely mapped circuits that allow memory allocation to each synapse in the module. Just as information is read directly back from the stored information in an SSD, in the case of the cortex this would be achieved via the MeshCODEs altering the synaptic transmissions passing through the column of memory cells after being called upon by the information-processing units.

### Hypothesis: An Addressable Read-Write Memory Mechanism

The need for memory storage in the brain to be highly ordered necessitates that the allocation of signal to each neuron in the network must be controlled by some form of memory allocation circuitry in the brain (this would seem to be a requirement whatever the method of memory storage). To enable data to be written to such a huge memory array, a detailed map of the memory architecture would be required to direct the flow of traffic through the memory circuits, to mechanically write data in specific synapses. Every neuron and synapse would be addressable by targeted communications. This process would require the brain equivalent of a file system architecture to ensure that new data was allocated to “free blocks” for writing but also that the data was stored in a logical, indexed way so that it could be retrieved.

The hippocampus plays a major role in learning and memory (O’Keefe and Dostrovsky, 1971) and has been identified as a key area of the brain for the processing of memory information. Studies have shown that initially memories might be stored in the hippocampus in some representative form and that these representations are later transferred into the cerebral cortex where the memory is stored as a long-lasting engram in neocortical networks (reviewed in Preston and Eichenbaum, 2013). This pipeline of data flow, i.e., collected by an input source, processed and stored transiently in the hippocampus before being written to long-term storage, would suggest that the hippocampus might contain the equivalent of a file system that maps out the data locations in the cortex and assigns information to specific cortical columns for long-term storage. In doing so, data would be given a specific address in memory, indexed and organised such that it is able to be retrieved and modified as and when required.

This transfer of data would be written by electrochemical signals transmitted through these main connections into the cortical memory blocks, which although they appear assigned at random to an outside observer, might instead be explicitly allocated by a memory controller to specific regions in the brain where that data is then stored. This data would be recorded by altering the pattern of 0s to 1s in specific MeshCODEs within that memory module. Strikingly, during such a writing process, almost no change would be visible in any of the synapses *per se* using current technology except for alterations in firing patterns, even though major changes in the data encoded in the synaptic MeshCODEs would be occurring. However, if seen at the molecular level this process would involve a whir of activity, with many changes in protein conformation as the data was written, transferred and transmitted across many synapses in the circuit.

### How Are Memories Retrieved So Quickly?

The brain can recall data incredibly fast, in less than a second, and this speed of access suggests that memory must be highly ordered. Neurons fire, and a lot of synaptic transmission occurs to achieve this feat, but it is a non-trivial task to store such vast amounts of information in a way that it is readily retrievable on demand. One way in which huge amounts of data can be organised to enable such rapid retrieval is to use a database. A database is an organised collection of data, arranged in a hierarchical storage structure that can be accessed, managed and updated. By indexing the data, it can all be stored and only looked up when required by going directly to the address of that data; this is not only much faster than holding everything in local memory, it also requires a smaller allocation of random access memory (RAM) when looking up data. Without a database and hierarchical storage structures, all data is given equal status, which means all data needs to be stored locally in case it is recalled. This near infinite number of possibilities makes the requirement for a hierarchical storage structure essential. If the memory storage in the cortex is like a huge database, then the hippocampus might also serve the role of a data management system. New memories would be processed and indexed prior to being written in specific locations in the cortical MeshCODEs.

### Hypothesis: A Role for Sleep in Data Management

Studies have shown that neuronal activity during sleep plays a central role in the transfer of memory representations from the hippocampus to the cortex (Lee and Wilson, 2002; Marshall et al., 2006; Buzsáki, 2015). Following a period of being awake, the brain has received a lot of information, some useful, some less so, and this needs to be processed and made coherent with the existing data. It is well established that sleep plays a central role in the consolidation of memory (Gais and Born, 2004; Stickgold, 2005; Walker, 2005; Marshall and Born, 2007; Draguhn, 2018). The physical nature of memory outlined here suggests that the reason for sleep might be the requirement to process huge amounts of data, integrate it with existing data, allocate it to, and physically write it into specific memory modules in the cortex for storage. Furthermore, as data is added, withdrawn and modified, overtime, indexes become fragmented which adversely affects performance. In a computer database, it is necessary to run index maintenance regularly and rebuild or reorganise indexes that require it. Rebuilding a database is an intensive process that involves unloading information out of the database, before reloading it back in a uniform, ordered fashion, optimising the filling space and re-indexing. In computers, such memory management processes are usually scheduled to be performed overnight when the data is not being used. The normal sleep cycle, cycling between deep sleep and rapid eye movement (REM) sleep (Carskadon and Dement, 2011), might perform such data management.

#### Deep Sleep

The initial deep sleep that occurs first in the normal sleep cycle might be required so that the brain can complete the calculations from that day. Sleep shuts down nearly all sensory inputs, maintaining only sufficient awareness for self-preservation. During periods of deep sleep, where brain activity is reduced, the lack of inputs thus might allow the system-level changes in the cytoskeleton and MeshCODE data storage to return to homeostasis, which ensures the data is correctly written and the newly formed memories are stabilised.

#### REM Sleep Might Be the Brain Actively Writing Data to the Cortex

Following deep sleep, the sleep patterns change and REM sleep begins. In this phase, the brain is very dynamic, with a lot of brain activity (Babloyantz et al., 1985; Purves et al., 2001; Boyce et al., 2016, 2017). It could be that REM is when the brain can now work on this new data, processing it and integrating it with the existing data. This requires extensive brain activity, stimulating the newly acquired data locations, moving old data around and writing new data to specific regions of the brain. During REM sleep, a lot of electrical activity points to something happening, but only little change in the structural properties of the brain would be apparent. However, on close inspection at the protein level, there would be a storm of activity as the coding is altered, the cytoskeletal machinery mediating communication mechanically through each neuron, talin-switches switching back and forth, and strings of information being used to signal to other neurons to drive the flow of information, altering the conformations and switch patterns in other synapses and in other neurons. The overall effect would be that data, the binary patterning of 0s and 1s, would be moved about, processed and written.

Following this reshuffling of the data, the brain goes back into a period of deeper sleep, perhaps to ensure that the new data patterns are established, and to allow the system time for further information-processing. Following memory stabilisation, another round of REM sleep occurs, allowing further data writing and reordering. This cycling between sleep states ideally occurs four to five times a night (Carskadon and Dement, 2011).

The result of this data management would see the transfer of newly encoded memories from the hippocampus to the cortex where they are consolidated and stabilised as long lasting MeshCODE patterns. These binary patterns, encoded in information-storing talin molecules, would represent the physical location of the engram, the hypothetical unit of cognitive information (Lashley, 1950; Josselyn and Tonegawa, 2020; Langille and Gallistel, 2020). The next day, the electrical fingerprint of that memory association would be different as the total number of neurons involved has been reduced as the memory was consolidated. The process would see each memory given a specific physical address, allowing it to be indexed so that it can be called whenever needed. A possible result of sleep deprivation could be that the brain’s memory soon becomes fragmented with deleterious effects on recall and performance.

A recent study showed that sleep-associated activity patterns can also erase memories from the hippocampus (Draguhn, 2018; Norimoto et al., 2018), suggesting a volatility to hippocampal data storage. The transient memory storage of the hippocampus thus is similar to RAM in computers, where data is volatile and only retained whilst powered on. This working memory would integrate new data with the existing data, and after the data is successfully processed and written to the cortex, the hippocampal MeshCODEs can be reset ready for the next day.

## Implications for Neurological Disease

For MeshCODE storage to work correctly, it would require each switch to unfold and refold with high fidelity. However, as the protein conformations that encode memory are located around the edge of the synapse, they might be susceptible to getting clogged up, and any disturbance of folding-refolding would corrupt the coding and scramble the information stored, leading to memory loss. Abnormal accumulation of amyloid-β and tau protein is linked to the memory loss and cognitive decline seen in Alzheimer’s disease (Brion, 1998) and amyloid-β has been proposed to disrupt the mechanical integrity of synapses leading to loss of memory (Woo et al., 2015). Part of this effect on memory might be that these tangles interfere with the MeshCODE directly, or that they perturb the mechanically ordered state of the integrin-talin-actin connections. Similarly, the loss of synaptic integrity associated with ageing (Morrison and Baxter, 2012; Mostany et al., 2013) would also result in loss of stored information. The MeshCODE framework might therefore provide a novel therapeutic axis for a number of synaptopathies (Grant, 2012), and neurodegenerative diseases such as Alzheimer’s (Dourlen et al., 2019) and dementia.

As a living machine, the mechanical coding of the brain would exist as a balance between opposing forces and factors. These forces are generated by the cells force-generating machinery that requires energy to function. Hypoxia (Graham, 1977) would quickly result in this machinery failing with the consequence that the synergy between the mechanical coding of the neurons is lost. As a result, once enough of the coding is corrupted it is unsalvageable so, even if the oxygen supply is re-established, the brain would not be able to recover this information and functioning once lost beyond a certain point.

## Conclusion and Perspectives

The MeshCODE theory presented here provides an original concept for the molecular basis of memory storage. I propose that memory is biochemical in nature, written in the form of different protein conformations in each of the trillions of synapses. This concept is based on the discovery of a complex network of mechanical switches in proteins like talin (Yao et al., 2016; Goult et al., 2018; Figure 2) that are built into the scaffolds of every synapse (Park and Goda, 2016; Lilja and Ivaska, 2018; Figure 3). These binary switches can be operated by the force-generation machinery of the cells cytoskeleton, offering a new view of the brain as a mechanical computer.

The capacity for storage of data in a binary form in each synapse identifies an addressable read-write memory mechanism, clearly pointing to a way, in which the brain might carry information forward in time and perform computation. Data written in binary, symbolic form would provide a basis for how the brain might function as an input-output system, in which its computation and data processing systems are founded on physical and mathematical principles (Gallistel and King, 2009). Remarkably, humankind’s efforts to produce optimal computation *in silico* may have led to architectures that bear a striking similarity to what nature might already have arrived at *in vivo*.

Sensory inputs are processed by the brain and trigger the appropriate motor responses as outputs allowing the animal to interact with the world. Action potential spike trains are well established as an organism’s way of sending information over long distances (Perkel et al., 1967; Strong et al., 1998; Fellous et al., 2004), similar to how electrical pulses carry information in electronic systems, yet quite how these voltage spikes that travel down axons carry information is not yet fully understood. In the MeshCODE framework proposed here, these spikes transfer information by altering the mechanical coding of both the sender and receiver cell. Diverse input signals, including visual, auditory, olfactory, temporal cues, self-movement (idiothetic), among others, are converted into electrical signals in the form of spike trains, and the precise patterns of these spikes trigger exact changes to the neurons. These changes include cytoskeletal alterations (Yao et al., 2006; Cingolani and Goda, 2008) which in the MeshCODE framework would update the switch patterns, such that the information the spike trains carry is integrated into the organism’s binary coding. This complex mechanical coding amounts to a machine code that is constantly running in all animals. From an initial state at birth, the life experiences and environmental conditions of the animal would be written into the code, creating a constantly updating, mathematical representation of the animal’s unique life. It is possible that consciousness is simply an emergent property arising from the interconnectedness of electrical signals connecting all these MeshCODEs, forming a complete mathematical representation of the world that gives rise to precise electrical signals that coordinate an entire biochemical organism in the context of its world.

The key to biochemical data storage would be the precise conformations of each mechanical switch in each and every synaptic adhesion. These conformations are mostly unmeasurable with existing technologies; using microscopy the talin visible in adhesions will not appear to change, even as the conformations of each alters during memory formation. However, as the size and composition of each synaptic adhesion complex will change in response to these altered patterns then observation of the adhesions themselves, identification of the ligands that engage them, and correlating these with the synapses activity should provide a readout of the process. Visualising these complexes is further complicated as any perturbation of the system will result in altered MeshCODE arrangements. However, the technical capabilities to observe protein states and forces acting on proteins in cells are advancing rapidly (Kumar et al., 2016; Ringer et al., 2017; Lemke et al., 2019) and used in conjunction with super-resolution microscopy techniques (Leterrier et al., 2017; Schnitzbauer et al., 2017; Jacquemet et al., 2020), optogenetic techniques (Liu et al., 2012), and the well-established strategies for studying neurotransmission (reviewed in Kandel et al., 2014) such conformational changes during memory formation should be detectable. Further, a number of talin-binding compounds have recently been identified (Yang et al., 2017; Bryce et al., 2019) and the effect of such compounds on learning and memory in animal systems might provide opportunities to pharmaceutically modulate these processes.

As a final comment, physical storage of memory would have significant potential future implications, not least that it might make the stuff of science fiction possible. If memory and consciousness are biochemical in nature, it is possible that one day we will fully decipher how the MeshCODE stores and computes information to form a mathematical representation of the world. In doing so we may not only understand the computations of the human mind, but also allow the transfer of the human mind from neural networks onto silicon chips running the human Operating System. A biochemical basis of memory storage also raises the possibility to not only read the memory of the living, but also the dead. Although short term memory might be accessible only transiently after death, long term MeshCODEs that are “write protected” might be possible to read for the duration of the integrity of the brain.

## Data Availability Statement

The original contributions presented in the study are included in the article/supplementary material, further inquiries can be directed to the corresponding author/s.

## Author Contributions

BG wrote the manuscript.

## Conflict of Interest

The author declares that the research was conducted in the absence of any commercial or financial relationships that could be construed as a potential conflict of interest.

## Footnotes

1 For the theory of the brain as a mechanical computer, the MeshCODE framework requires that there are mechanical binary switches present in each synapse that can be controlled by synaptic transmission. Whilst this article focuses on the mechanical switches of talin as the minimal unit of memory, there are other mechanosensitive proteins, such as vinculin, filamin, p130Cas and α-catenin [the cell adhesion molecules at synapses are reviewed in Kilinc (2018)], that might also store information and form part of the MeshCODE.
